# The Combination of *Buchloe dactyloides* Engelm and Biochar Promotes the Remediation of Soil Contaminated with Polycyclic Aromatic Hydrocarbons

**DOI:** 10.3390/microorganisms12050968

**Published:** 2024-05-11

**Authors:** Yuancheng Wang, Ao Li, Bokun Zou, Yongqiang Qian, Xiaoxia Li, Zhenyuan Sun

**Affiliations:** 1 Research Institute of Forestry, Chinese Academy of Forestry, Beijing 100091, China; evs1226@163.com (Y.W.); liao11100@outlook.com (A.L.); 2 Institute of Ecological Conservation and Restoration, Chinese Academy of Forestry, Beijing 100091, China; zoubk@caf.ac.cn (B.Z.); qianyq@caf.ac.cn (Y.Q.)

**Keywords:** Polycyclic aromatic hydrocarbons, *Buchloe dactyloides*, biochar, phytoremediation, microorganism

## Abstract

Polycyclic aromatic hydrocarbons (PAHs) cause serious stress to biological health and the soil environment as persistent pollutants. Despite the wide use of biochar in promoting soil improvement, the mechanism of biochar removing soil PAHs through rhizosphere effect in the process of phytoremediation remain uncertain. In this study, the regulation of soil niche and microbial degradation strategies under plants and biochar were explored by analyzing the effects of plants and biochar on microbial community composition, soil metabolism and enzyme activity in the process of PAH degradation. The combination of plants and biochar significantly increased the removal of phenanthrene (6.10%), pyrene (11.50%), benzo[a]pyrene (106.02%) and PAHs (27.10%) when compared with natural attenuation, and significantly increased the removal of benzo[a]pyrene (34.51%) and PAHs (5.96%) when compared with phytoremediation. Compared with phytoremediation, the combination of plants and biochar significantly increased soil nutrient availability, enhanced soil enzyme activity (urease and catalase), improved soil microbial carbon metabolism and amino acid metabolism, thereby benefiting microbial resistance to PAH stress. In addition, the activity of soil enzymes (dehydrogenase, polyphenol oxidase and laccase) and the expression of genes involved in the degradation and microorganisms (*streptomyces*, *curvularia*, *mortierella* and *acremonium*) were up-regulated through the combined action of plants and biochar. In view of the aforementioned results, the combined application of plants and biochar can enhance the degradation of PAHs and alleviate the stress of PAH on soil microorganisms.

## 1. Introduction

Polycyclic aromatic hydrocarbons (PAHs) are aromatic compounds which widely distributed in soil and produced in large quantities by industrial activities that are hazardous to human beings and extremely difficult to degrade [1]. Previous studies have demonstrated that PAHs can diminish soil fertility and quality by impacting the nutrient cycle, including carbon and nitrogen [2]. The remediation of PAHs in soil largely depends on indigenous microorganisms [3], whereas PAHs have a negative effect on soil microorganisms by inhibiting soil metabolic processes [2]. This may adversely affect the removal of PAHs. Therefore, it is important to clarify the effects of different treatments on the PAHs adaptability of indigenous microorganisms.

Phytoremediation is an environmentally-friendly remediation method that accelerates the degradation of PAHs through rhizosphere processes. Nutrient cycling, physical and chemical properties of soil will be affected by roots to form a unique rhizosphere environment [4]. Furthermore, plants can shape rhizosphere microorganisms by actively or passively releasing root exudation that act as a carbon source or a signal for microorganisms, recruiting specific beneficial microorganisms and altering the composition of indigenous microorganisms [5,6]. Other studies have determined that plants also engage in competition with microorganisms with microorganisms for soil nutrients, thereby influencing the functionality of microorganisms [7]. This may explain why the degradation efficiency of PAH remains unchanged or decreases under phytoremediation. Therefore, exploring the effect of plant roots on soil microorganisms is very important for the removal of PAHs.

Soil additives have widely emerged as a treatment for degrading pollution in recent years [8]. Additives can significantly affect soil remediation by improving the soil environment due to their own characteristics, such as rich nutrient elements and special structure [9]. Among these, biochar has been found to enhance remediation efficiency by modulating soil microbial structure and improving microbial metabolism due to its abundant nutrient content [9,10]. Moreover, biochar, as a potential source of soil PAHs [11], may also reduce the bioavailability of PAHs [12]. Although the feasibility of the combined application of phytoremediation and biochar is still controversial, it has been proved that biochar can improve the reduced soil fertility and soil metabolism under stress [13,14]. In addition, biochar applied may affect the composition of root exudates and the root morphology, such as increasing the content of organic acids and root area [15]. The elevated concentration of organic acids could potentially facilitate the degradation of PAH [16]. Therefore, biochar may promote the rhizodegradation of PAH by changing the root exudation strategy. Hence, when biochar and plants combine for remediating PAH contaminated soil, they can mediate the function of soil microorganisms and establish intricate interactions with microorganisms during the microbial degradation process of PAH, and ultimately affect the bioremediation. However, adverse results have been observed in studies of biochar remediation for the remediation of PAH contamination [17]. This may be due to the fact that biochar not only stimulates microbial biodegradation but also competes for the nutrients needed for rhizosphere microbial growth while stimulating the growth of plant roots [18,19,20]. This shows that the complex interaction between plants and microorganisms will be affected by biochar and ultimately promote or inhibit the adaptation of soil microorganisms to PAHs stress. And the adaptation of microorganisms to PAHs will affect the removal of PAHs in soil. However, the existing studies have not systematically studied the PAHs degradation and adaptability of microorganisms. Therefore, it is urgent to fully clarify the effects of biochar on PAHs degradation, microorganisms and rhizosphere soil environment, thereby assess the adaptability and degradation of biochar and plants to microorganisms to PAHs.

The resistance of microorganisms to PAH stress can be clarified, and the role of microorganisms in PAH degradation can be revealed by analysis of soil enzyme activity, microbial structure and function combined with soil metabolism. *Buchloe dactyloides* is a highly tolerant herbaceous plant [21]. In this study, we conducted 60-day experiment on potted plants in greenhouse to clarify the mechanism of soil remediation for PAH pollution and the effects on soil adaptability by combining *B. dactyloides* and biochar. The following hypotheses were addressed: (1) The combination of *B. dactyloides* roots and biochar stimulate microorganisms involved in PAH degradation, including changing the structure and functional patterns of microorganisms. (2) *B. dactyloides* roots and biochar can improve soil health and metabolism. This study may provide guidance for the remediation of PAH contaminated soil by plants combined with biochar.

## 2. Materials and Methods

### 2.1. Pot Experiment

The soil was collected at depths of 0–20 cm from an agricultural field in Beijing, China (40°0′27″ N, 116°15′22″ E). The properties of soil and biochar were detailed in the Tables S1 and S2. The PAHs contaminated soil containing phenanthrene (Phe), pyrene (Pyr) and benzo(a)pyrene (Bap), with or without 1% of biochar addition, was prepared. Preparation of PAHs-contaminated soil was performed as previously reported [14].

Three *B. dactyloides* were transferred to a pot full of 0.7 kg soil and placed under natural light at a temperature of 25–35 °C. The following four treatments were utilized: PAH contaminated soil (N); PAH contaminated soil with biochar (B); PAH contaminated soil with *B. dactyloides* (P); PAH contaminated soil with *B. dactyloides* and biochar (PB). The field capacity of soil was maintained at 60% by regularly weighing the pots and adding distilled water. The position of the pot changes randomly every week. After 60 days of incubation, the loose soil attached to the roots was removed by shaking roots vigorously, and the rhizosphere soil was carefully collected with a sterilized brush. Additionally, non-rhizosphere soil was also collected. The soil samples were sieved (2-mm mesh). Soil was stored in liquid nitrogen for further analysis. The soil properties after treatment were detailed in the Table S3.

### 2.2. Determination of PAHs

Freeze-dried soil (Christ-Alpha 1–4 LD plus) was subjected to ultrasonic extraction with a 30 mL mixture of n-hexane and acetone (2:1, *v*/*v*). The contents of Phe, Pyr and Bap in soil were quantitatively analyzed by the internal standard method [22]. Determination of PAHs content by Agilent HP 7890 gas chromatograph combined with Agilent HP 5975C inert mass selective detector (7890/5975C).

### 2.3. Determination of Enzyme Activity

The soil polyphenol oxidase (PPO) activity was measured using the pyrogallol colorimetric method. Soil dehydrogenases (DHA) activity was measured as previously reported [23]. Soil laccase activity was determined by measuring the oxidation of ABTS. The soil catalase (CAT) activity was measured using a soil catalase activity assay kit (Solarbio, Beijing, China). The soil urease activity was determined by using the indophenol blue colorimetry method.

### 2.4. High-Throughput Sequencing and Quantitative Polymerase Chain Reaction (qPCR)

The DNA extraction, Illumina sequencing and qPCR were carried out with reference to previous reports [22]. Soil DNA was extracted from 0.5 g of each soil sample using the E.Z.N.A. Soil DNA Kit (Omega Biotek, Norcross, GA, USA) according to the manufacturer’s protocol. 16S rRNA and ITS genes of distinct regions (16S V3–V4, ITS1) were amplified using specific primers with the barcode. The primer pairs and standard curve of 16S and ITS qPCR genes were shown in Supporting Information (Table S4, Figure S1).

### 2.5. Soil Metabolite Assay

Soil metabolites were extracted and determination was performed as previously reported [24,25,26]. The LC analysis was performed on a Vanquish UHPLC System (Thermo Fisher Scientific, Waltham, MA, USA). Chromatography was carried out with an ACQUITY UPLC ^®^ HSS T3 (150 × 2.1 mm, 1.8 µm) (Waters, Milford, MA, USA). The column maintained at 40 °C. The flow rate and injection volume were set at 0.25 mL/min and 2 μL, respectively. For LC-ESI (+)-MS analysis, the mobile phases consisted of (C) 0.1% formic acid in acetonitrile (*v*/*v*) and (D) 0.1% formic acid in water (*v*/*v*). Separation was conducted under the following gradient: 0~1 min, 2% C; 1~9 min, 2~50% C; 9~12 min, 50~98% C; 12~13.5 min, 98% C; 13.5~14 min, 98~2% C; 14~20 min, 2% C. For LC-ESI (−)-MS analysis, the analytes was carried out with (A) acetonitrile and (B) ammonium formate (5 mM). Separation was conducted under the following gradient: 0~1 min, 2%A; 1~9 min, 2~50%A; 9~12 min, 50~98%A; 12~13.5 min, 98%A; 13.5~14 min, 98~2%A; 14~17 min, 2%A.

Mass spectrometric detection of metabolites was performed on Orbitrap Exploris 120 with ESI ion source. Simultaneous MS1 and MS/MS (Full MS-ddMS2 mode, data-dependent MS/MS) acquisition was used. The parameters were as follows: sheath gas pressure, 30 arb; aux gas flow, 10 arb; spray voltage, 3.50 kV and −2.50 kV for ESI(+) and ESI(−), respectively; capillary temperature, 325 °C; MS1 range, *m*/*z* 100–1000; MS1 resolving power, 60,000 FWHM; number of data dependant scans per cycle, 4; MS/MS resolving power, 15,000 FWHM; normalized collision energy, 30%; dynamic exclusion time, automatic.

### 2.6. Date Analysis

The normality of the datasets was tested using SPSS 22.0 (SPSS Inc., Chicago, IL, USA). The variables were analyzed using two-way analysis of variance, and using the statistical package IBM SPSS Statistics software (SPSS 22.0). The co-occurrence network, non-metric multidimensional scaling (NMDS), PICRUSt (KEGG, http://www.kegg.jp/, accessed on 17 April 2023), mantel test and metabolomics date analysis were measured using a modified method [2,22]. Linear discriminant analysis effect size (LEfSe) was utilized to identify significant microbial responders (LDA > 3.0). Adonis, Anosim, environmental niche width, Variance Partitioning Analysis (VPA) and Envfit analysis were conducted using the retatix R packages. Envfit analysis was used to determine the correlation between environment, microbial communities and KEGG.

## 3. Results

### 3.1. Degradation Rate of PAHs and Enzymatic Activity

**Figure 1:**
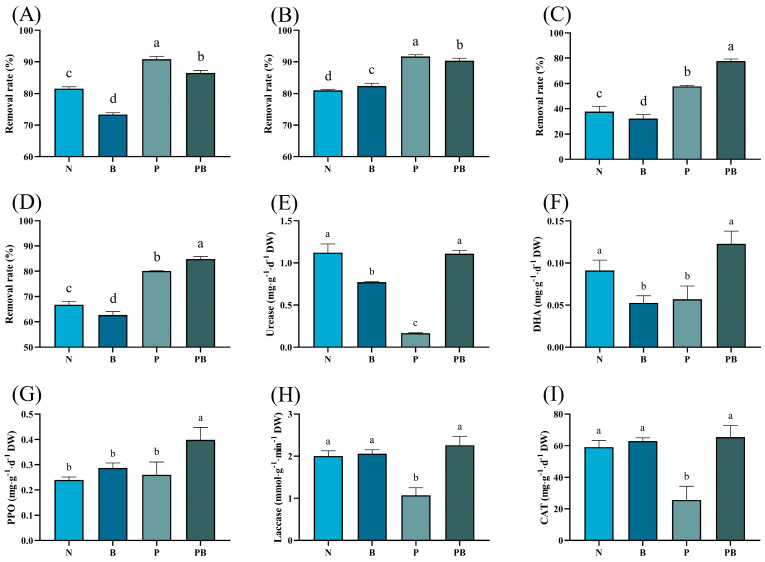
The removal rate of Phenanthrene (**A**), Pyrene (**B**), Benzo[a]pyrene (**C**) and total PAHs (**D**) in different treatment. Effect of different bioremediation treatments on soil enzyme activities, including urease (**E**), DHA (**F**), PPO (**G**), laccase (**H**) and CAT (**I**). N: natural attenuation; B: biochar remediation; P: phytoremediation; PB: plant−biochar remediation. Averages ± SE were listed (*n* = 3). Different letters indicate that values are significantly different (*p* < 0.05).

There were significant differences in the removal rate of PAHs under different treatments (Figure 1A–C). The degradation efficiencies of three kinds of PAHs in the B treatment were lower than N treatment. The degradation rate of PAHs in the rhizosphere was significantly higher than that in the N treatment. The combined application of *B. dactyloides* and biochar brought the highest removal rate of Bap and total PAHs. The activities of urease and DHA under N and PB treatments were higher than those under other treatments. The activity of PPO was the highest in rhizosphere soil containing biochar. Under P treatment, the activities of laccase and CAT were lowest.

### 3.2. Shifts in Microbial Community and qPCR

**Figure 2:**
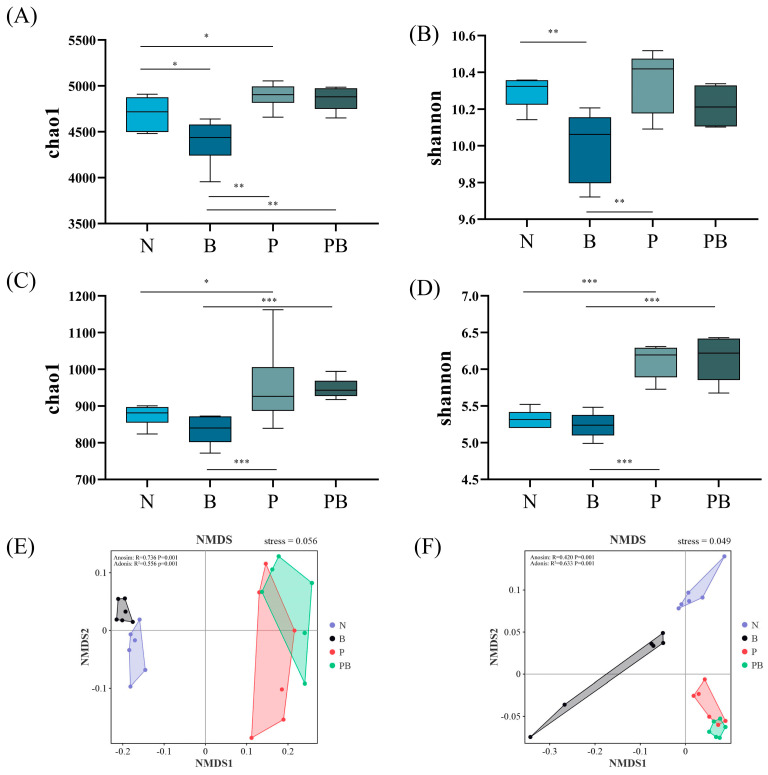
Chao1 and Shannon indices of the bacterial (**A**,**B**) and fungal (**C**,**D**). NMDS of the bacterial (**E**) and fungal (**F**). N: natural attenuation; B: biochar remediation; P: phytoremediation; PB: plant−biochar remediation. The significance of the test was analyzed by Adonis and Anosim. *, *p* < 0.05; **, *p* < 0.01; ***, *p* < 0.001.

**Figure 3:**
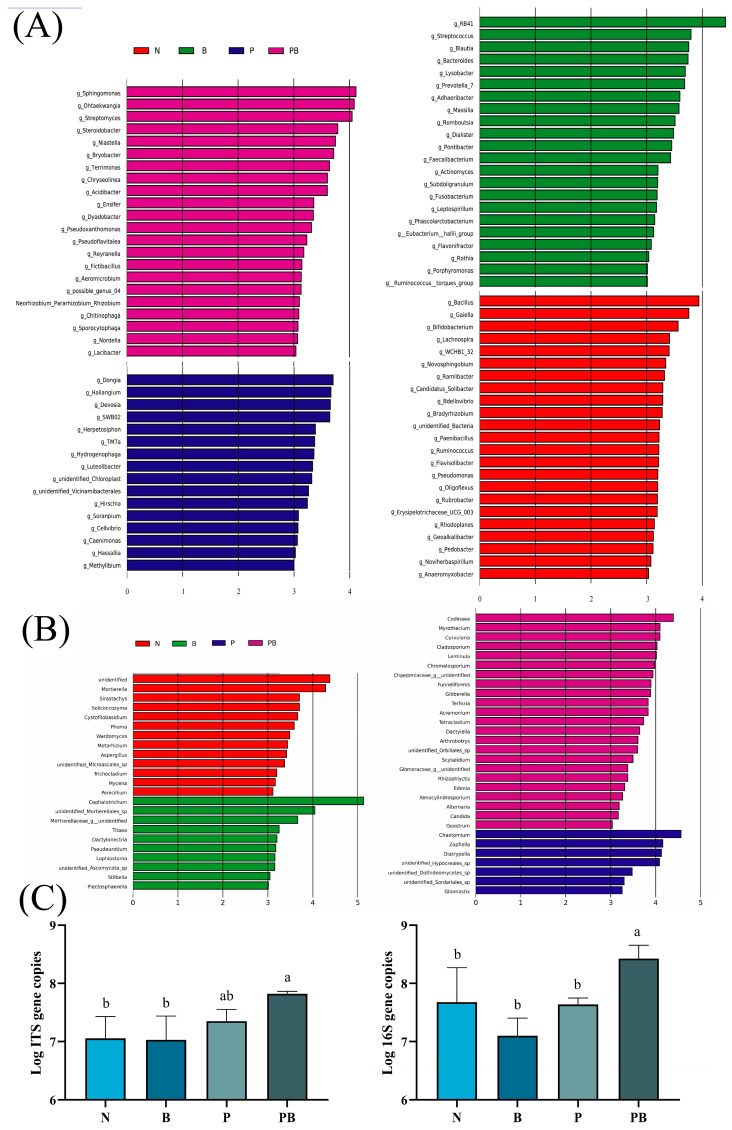
Chicken breed and line-specific biomarkers. LEfSe analysis shows differentially abundant genera of bacteria (**A**) and fungi (**B**) as biomarkers determined using Kruskal-Wallis test (*p* < 0.05) and Wilcoxon test (*p* < 0.05) with LDA > 3.0. Gene copy numbers of 16S and ITS in different treatments (**C**). N: natural attenuation; B: biochar remediation; P: phytoremediation; PB: plant−biochar remediation. Averages ± SE were listed (*n* = 3). Different letters indicate that values are significantly different (*p* < 0.05).

The chao1 index of microbial community and the shannon index of fungal community in rhizosphere soil were significantly higher than those in non-rhizosphere soil (Figure 2A–D). This indicated that the abundance and diversity of the rhizosphere microbial community were higher. NMDS showed that different treatments affected the similarity of microbial communities (Figure 2E,F). Lefse analysis showed that there were significant differences in the biomarkers of microbial groups under different treatments. *Bacillus.g*, *Novosphingobium.g*, *Paenibacillus.g* and *Pseudomonas.g* were the main bacterial participants in PAH degradation under N treatment, while *Streptococcus.g* and *Massilia.g* were the main bacterial participants in PAH degradation under B treatment. Moreover, *Methylibium.g*, *Devosia.g* and *Hydrogenophaga.g* were the main bacterial participants in the degradation of PAHs under P treatment, while *Pseudoxanthomonas.g*, *Streptomyces.g*, *Sphingomonas.g* and *Ensifer.g* were the main participants in the degradation of PAHs under PB treatment (Figure 3A). *Mortierella.g*, *Phoma.g*, *Aspergillus.g* and *Trichocladium.g* were the main fungal participants of PAH degradation under N treatment, while *Chaetomium.g* was the main fungal participant of PAH degradation under P treatment. In addition, *Curvularia.g*, *Cladosporium.g* and *Acremonium.g* were the main fungal participants of PAH degradation under PB treatment (Figure 3B). In the PB group, the bacterial biomass was the highest, and the fungal biomass was higher than that of N and B treatments (Figure 3C).

### 3.3. Environmental Factors and Function of Microbial Community

**Figure 4:**
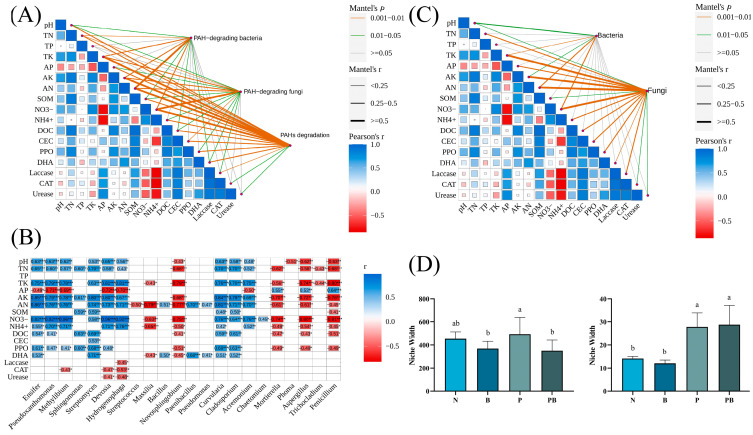
(**A**) Mantel tests between PAHs and soil property. (**B**) Correlation coefficients among soil property and PAH-degrading microorganism. (**C**) Mantel tests between microorganism and soil property. (**D**) The environmental niche width of bacterial (left) and fungal (right) under different treatments. N: natural attenuation; B: biochar remediation; P: phytoremediation; PB: plant−biochar remediation. Averages ± SE were listed (*n* = 6). Different letters indicate that values are significantly different (*p* < 0.05). *, *p* < 0.05; **, *p* < 0.01; ***, *p* < 0.001.

**Figure 5:**
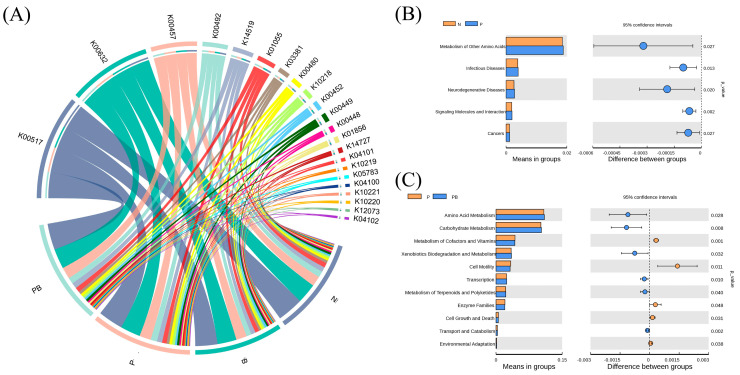
(**A**) Alters in function profiles of microorganisms under different groups. Predicted relative abundance of bacterial metabolic functions in the N vs. P treatment (**B**) and P vs. PB treatment (**C**). N: natural attenuation; B: biochar remediation; P: phytoremediation; PB: plant−biochar remediation. Averages ± SE were listed (*n* = 6).

**Figure 6:**
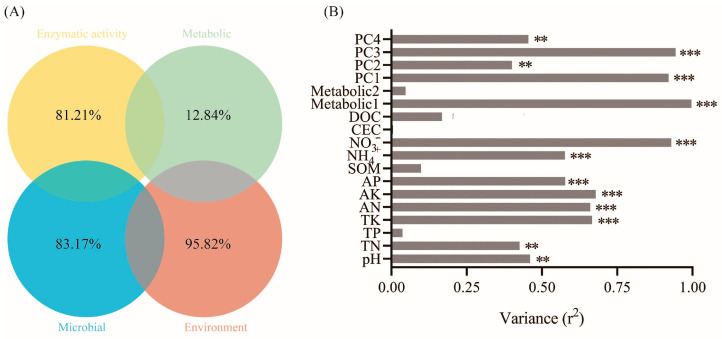
(**A**) Venn showing the results of VPA (adjusted R2 value) for PAH degradation explained by enzymatic activity, metabolic, microbial and environmental properties categories. (**B**) Bacterial function profiles of KEGG (PAHs degradation genes) explained by metabolic, metabolic and environmental variables based on the Envfit analysis. **, *p* < 0.01; ***, *p* < 0.001.

Mantel test and Spearman models were used to screen the soil factors related to PAH degradation and PAH degrading microorganisms (Figure 4A–C). TN, TK, AK, AN and NO_3_^−^ may be the main factors potentially involved in the degradation of PAHs. Among them, TK, AN and NO_3_^−^ were significantly correlated with microorganisms. There were significant differences in niche breadths of soil microbial bacteria and fungi communities under different treatments (Figure 4D). The niche breadth of rhizosphere bacteria under biochar conditions was lower than P treatment, which indicated that the bacterial community tended to be specialized species. PICRUSt was used to predict the biodegradation intensity of genes involved in PAH degradation (Figure 5A). The expression levels of PAH degradation gene in PB treatment was significantly higher than that in other treatments. Bacterial functions in amino acid, carbohydrate, terpene, and flavonoid metabolism, as well as exogenous biodegradation and metabolism were significantly improved under PB treatments (Figure 5B). And soil microflora, soil metabolism and soil environment were significantly related to microbial function (Figure 6B). Finally, VPA showed that the difference in PAH removal rate was mainly attributed to soil microflora, soil metabolism, soil environment and microbial function (Figure 6A).

### 3.4. Soil Metabolism and Co-Occurrence Network Analysis

**Figure 7:**
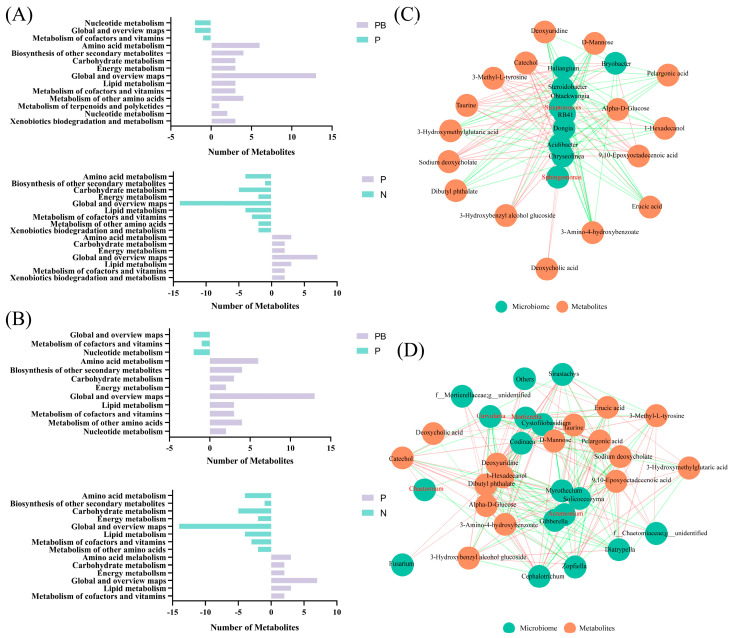
Differential metabolite-based metabolic pathways in bacteria (**A**) and fungi (**B**) under different treatments. Network of co−occurring different bacteria (**C**) and fungi (**D**) with different metabolites. The red line is a positive correlation and the green is a negative correlation. The thickness of the line represents the correlation coefficient. N: natural attenuation; P: phytoremediation; PB: plant−biochar remediation.

The differential metabolite pathway showed that the combined application of biochar and plants significantly improved the main metabolic processes in PAH contaminated soil. Compared with P treatment, the amino acid metabolism, secondary metabolite biosynthesis, terpenoid and flavonoid metabolism, carbohydrate metabolism, and lipid metabolism of the bacterial community under PB treatment exhibited significant improvements. Furthermore, the secondary metabolite biosynthesis and amino acid metabolism of the bacterial community under P treatment were significantly improved compared with N treatment (Figure 7A). Compared with P treatment, the amino acid metabolism, secondary metabolite biosynthesis, carbohydrate metabolism and lipid metabolism of fungal community under PB treatment were significantly improved. The secondary metabolite biosynthesis and amino acid metabolism of fungal community under P treatment were significantly improved compared with N treatment (Figure 7B).

For the soil bacterial community, *Sphingomonas.g*, *Streptomyces.g*, *Bacillus.g*, which may be involved in the degradation of PAHs, are the core bacteria. Theobromine, 3-Hydroxybenzyl alcohol glucoside and 9, and 10-Epoxyoctadecenoic acid are the metabolites with significant positive correlation with *Sphingomonas.g*, *Streptomyces.g* (Figure 7C). *Acremonium.g*, *Mortierella.g*, *Chaetomium.g*, *Curvularia.g* are fungi involved in PAH degradation and constitute the core fungal community. Catechol, Gingerol, Quercetin, 3-Hydroxybenzyl alcohol glucoside and 9, 10-Epoxyoctadecenoic acid are the metabolites with significant positive correlation with *Mortierella.g*, *Curvularia.g*, *Chaetomium.g* (Figure 7D).

## 4. Discussion

### 4.1. The Effects of Biochar and Plant Roots on PAH Contaminated Soil and Microorganisms

In this study, the application of biochar increased the removal rate of Pyr, while still decreased the removal of Phe and Bap, which is different from the results of other studies [14]. This discrepancy may be attributed to variations in the physical and chemical properties of biochar. The difference in removal of Phe and other PAHs by biochar may be explained as the variation in bioavailability caused by the number of benzene rings and the bond angle of ring [17]. P treatment and PB treatment significantly increased the removal rate of PAHs, which indicated that phytoremediation was an attractive approach to remove PAHs from soil. PAH-degrading enzyme activity is generally considered to reflect the ability of PAH degradation [27]. In this study, the activity of DHA, PPO, laccase involved in PAH degradation was the highest in PB treatment, and they were significantly related to the degradation rate of PAH (Figure 4A). The removal rate of Bap increased significantly under PB treatment may be attributed to the increase in PPO activity contributing to the degradation of high molecular weight PAH [28,29]. Moreover, the activities of urease and CAT increased under PB treatment, reflecting the enhancement of soil nitrogen use efficiency and the improvement of soil health. The decrease in CAT, DHA, laccase, and urease activities in the rhizosphere soil may be attributed to the competition between *B. dactyloides* and indigenous microorganisms for nutrients such as carbon and nitrogen, and the combined treatment reversed this negative effect (Table S3). Our investigation revealed a substantial increase in microbial biomass in PAH contaminated soil as a result of the synergistic effect of *B. dactyloides* and biochar (Figure 3C). Thus, *B. dactyloides* and biochar increased microbial metabolic activity, enhanced microbial PAH degradation ability and alleviated PAH stress.

The structural composition of microorganisms is considered to be one of the main drivers of PAH dissipation [30,31]. *Pseudoxanthomonas.g*, *Streptomyces.g*, *Sphingomonas.g*, *Ensifer.g*, *Methylibium.g*, *Devosia.g*, *Hydrogenophaga.g*, *Bacillus.g*, *Paenibacillus.g*, *Pseudomonas.g*, *Curvularia.g*, *Cladosporium.g*, *Acremonium.g* and *Chaetomium.g* have been identified as potential participants in the degradation of PAH. We found that biochar and *B. dactyloides* roots induced soil microbial community reconstruction (Figure 2E,F). The difference between different treatments may be due to soil properties. TN, pH, TK, AK, AN, NO3-, SOM and DOC were identified as soil factors related to the change of microbial community structure and PAH degradation (Figure 4A). In this study, biochar and *B. dactyloides* increased soil nutrient content, while the increased nutrient content increased the bioavailability of PAHs, and may promote the biodegradation of PAHs through co-metabolic pathway. *Pseudoxanthomonas.g*, *Sphingomonas.g*, *Streptomyces.g* and *Ensifer.g* were significantly enriched as PAH degrading bacteria in rhizosphere soil with the presence of biochar. Biochar provided protection for *Sphingomonas.g* that can utilize PAH as carbon source [32], thus promoting the degradation of PAH under the combined application of plants and biochar. The enrichment of *Pseudoxanthomonas.g* may be attributed to the enrichment of soil nutrients subsequent to biochar application [33], potentially leading to the promotion of NH4^+^-N transformation and nitrogen fixation along with *Ensifer.g* (Table S3) [34,35]. The increase of soil PAH removal rate could be ascribed to root exudation promoting the degradation of soil PAH by *Streptomyces.g* [36]. *Devosia.g*, *Hydrogenophaga.g*, and *Methylibium.g* mainly contributed to the degradation of low molecular weight PAH [37,38,39], which supported the significantly increased Phe and Pyr removal in the rhizosphere of *B. dactyloides* under P treatment. The biodegradation intensity of PAH is related to the abundance and composition of genes. Dioxygenase, dehydrogenase, and hydroxylase genes are directly involved in the oxidative degradation of PAH [40,41]. The proportions of functional genes related to dioxygenase, dehydrogenase and hydroxylase in different treatments was consistent with the degradation of PAHs (Figure 5A). Consistent with previous studies [10,22], this suggests that key bacteria and functional genes played a pivotal role in soil PAH degradation, and this may explain the increase in removal rate of PB treatments.

### 4.2. The Effects of Biochar and Plant Roots on Soil Carbon Metabolism Associated with PAH Degradation

Microbial metabolism often reflects soil microbial community and function in response to stress. As an organic pollutant, PAH can inhibit the soil microbial activity, alter the community structure, function and metabolic activities of microorganisms, ultimately affect the soil quality [2]. Plants and biochar usually have different strategies for regulating microbial metabolism. In this study, no significant improvement in metabolic activity of microorganisms under P treatment (Figure 7A,B). Biochar amendment significantly improved the microbial carbon metabolism and amino acid metabolism in rhizosphere soil (Figure 7A,B), and specialized soil niche (Figure 4D), which was beneficial for microorganisms to adapt to PAH stress. This may be due to the fact that biochar improved the soil environment and affects the recruitment of rhizosphere microorganisms by altering the composition of root exudation [42]. In addition, the increased organic acid metabolites can effectively improve the bioavailability and promote the biodegradation of PAH by releasing PAH bound in organic matter through modifications to the rhizosphere enhanced with biochar [43]. The improvement of microbial amino acid metabolism by *B. dactyloides* roots and biochar may also favor the expression of PAH degradation genes [44]. And the involvement of amino acid metabolism in microbial detoxification reflects the improvement of microbial tolerance [10]. Similarly, the combination of *B. dactyloides* roots and biochar improved PAH bioavailability by significantly increasing lipid metabolism. Intermediate metabolites produced by indigenous microorganisms involved in the degradation of PAH, especially high molecular weight PAH, are components of the soil carbon cycle [45]. Therefore, the promotion of microbial carbon metabolism is beneficial for the in-situ degradation of PAH.

Biochar promoted soil carbon metabolism and bioremediation by increasing the contents of soil organic carbon [46]. Some soil metabolites and root exudation participate in the process of PAH degradation through the co-metabolic pathway [47]. Therefore, biochar could affect the in-situ degradation of PAH by regulating the utilization of soil carbon resources and changing soil metabolites. Moreover, soil metabolism affects microbial function together with microbial structure and soil characteristics (Figure 6B). This shows that there are complex interactions among bacteria, fungi, soil metabolites and biodegradation. Metabolic intermediates (catechol, dibutyl phthalate and 3-amino-4-hydroxybenzoate), root exudates (deoxycholic acid and taurine), coexisted with microorganisms involved in PAH degradation (*streptomyces*, *curvularia*, *mortierella* and *acremonium*) (Figure 7C,D) [48]. This suggests that plants and microorganisms forming complex interactions under PAHs stress jointly participate in and influence the removal of PAHs from soil. And it indicates that these compounds may be involved in the degradation of PAHs as co-metabolic substrates. This suggests that these compounds may be involved in PAH degradation as co-metabolic substrates. In addition, VPA showed that the significant improvement of PAH removal rate under PB treatment was driven by soil enzyme activity, soil metabolism, microflora and soil characteristics. Our research shows that biochar and *B. dactyloides* as remediation methods for PAHs contaminated soil can significantly improve the removal rate of PAHs, soil environment and the adaptability of microorganisms to PAHs.

## 5. Conclusions

*B. dactyloides* and biochar improved the removal rate of PAH. *B. dactyloides* and biochar increased soil enzyme activity, affected soil environment, regulated soil metabolism, changed the structure and function of soil microorganisms, in which the changes of soil environment, the structural composition of microorganisms and the expression of functional genes were considered to be the main driving forces of PAH removal. In addition, the microbial activity was increased, and the microbial carbon metabolism and amino acid metabolism were improved to cope with PAH stress under the combined action of *B. dactyloides* and biochar. These findings are of great significance for elucidating the underlying mechanism of this bioremediation strategy.

## Data Availability

We will upload the data to the platform and provide web pages before the article is published.

## Supplementary Materials

The following supporting information can be downloaded at: https://www.mdpi.com/article/10.3390/microorganisms12050968/s1. Table S1: Properties of soil in this study. Table S2: Main physiochemical characteristics and PAHs concentrations of woody biomass biochar pyrolyzed at 400 °C. Table S3: Properties of soil in this study. Table S4 Primers used for qPCR. Figure S1: Standard curves of 16S (a) and ITS (b).
