# Supplementary material for: The Combination of Buchloe dactyloides Engelm and Biochar Promotes the Remediation of Soil Contaminated with Polycyclic Aromatic Hydrocarbons
Source: Microorganisms. 2024 May 11;12(5):968. doi: 10.3390/microorganisms12050968 (PMC11124401; doi:10.3390/microorganisms12050968)
Supplement: Supplementary file 1 [file microorganisms-12-00968-s001.zip › microorganisms-2958559-supplementary.pdf]

Table S1 Properties of soil in this study

	pH	SOM	TN	TP	TK	AP	AK	AN	DOC	NH_4^+	NO_3^-	CEC
		—— g kg^{-1} ——				—— mg kg^{-1} ——						c mol kg^{-1}
Content	8.42± 0.08	18.86± 0.51	0.93± 0.01	0.94± 0.06	18.03± 0.63	35.99± 0.59	173.78± 17.43	101.52± 2.79	100.14± 4.56	31.44± 0.22	6.19± 0.19	11.53± 0.34

Control: a PAH-stressed soil. SOM: soil organic matter; TN: total nitrogen; TP: total phosphorus; TK: total potassium; AP: available phosphorus; AK: available potassium; AN: Alkali-hydrolysable nitrogen; DOC: dissolved organic carbon; NH_4^+: ammonia nitrogen; NO_3^-: nitrate nitrogen; CEC: cation exchange capacity.

Table S2 Main physiochemical characteristics and PAHs concentrations of woody biomass biochar pyrolyzed at 400 °C.

	Content		Content
BET surface area (m^2/ g)	2.88	pH	7.60
Dissolved organic matter (g/ kg)	1.14	C (%)	81.07
Cation exchange capacity (mmol/ kg)	9.74	H (%)	3.93
Moisture content (%)	1.87	O (%)	8.36
Particle size (%; ≥ 1 mm)	8.90	N (%)	0.41
Particle size (%; 0.5-1 mm)	31.85	H/C	0.58
Particle size (%; 0.25-0.5 mm)	22.59	O/C	0.08
Particle size (%; 0.1-0.25 mm)	14.46	(O+N)/C	0.14
Particle size (%; 0.075-0.1 mm)	4.53	Phenanthrene (μg/ kg; DW)	99.6 ± 39.5
Particle size (%; <0.075 mm)	17.66	Pyrene (μg/ kg; DW)	15.9 ± 4.6
Porosity ratio (%)	20.55	Benzo(a)pyrene (μg/ kg; DW)	nd
Ash content (%)	6.12		

DW: Dry weight

Table S3 Properties of soil in this study

Group	pH	SOM	TN	TP	TK	AP	AK	AN	DOC	NH_4^+	NO_3^-	CEC
		—— g kg^{-1} ——				—— mg kg^{-1} ——						c mol kg^{-1}
N	8.37± 0.46	16.74± 0.56	0.87± 0.04	0.88± 0.00	18.03± 0.63	35.67± 0.54	173.93± 9.55	91.37± 7.03	83.32± 3.69	3.37± 0.23	14.66± 0.66	12.02± 0.42
B	8.39± 0.31	20.42± 0.51	1.01± 0.02	0.89± 0.01	18.03± 0.63	34.37± 0.98	193.36± 8.50	79.11± 2.26	91.60± 2.12	3.07± 0.19	15.55± 0.53	12.13± 0.19
P	8.41± 0.10	16.64± 0.20	0.98± 0.03	0.88± 0.04	18.03± 0.63	32.03± 0.33	212.64± 4.99	97.64± 1.82	90.64± 1.20	4.29± 0.13	22.02± 1.73	11.59± 0.09
PB	8.40± 0.06	23.20± 0.05	1.17± 0.02	0.89± 0.02	18.03± 0.63	33.87± 0.60	254.69± 4.73	107.19± 1.17	105.52± 1.37	3.66± 0.06	20.86± 0.14	13.11± 0.13

SOM: soil organic matter; TN: total nitrogen; TP: total phosphorus; TK: total potassium; AP: available phosphorus; AK: available potassium; AN: Alkali-hydrolysable nitrogen; DOC: dissolved organic carbon; NH_4^+: ammonia nitrogen; NO_3^-: nitrate nitrogen; CEC: cation exchange capacity.

Table. S4 Primers used for qPCR.

Target group	Primers	Sequences (5′ to 3′)
16S	341F	CCTAYGGGRBGCASCAG
	806R	GGACTACNNGGGTATCTAAT
ITS	ITS5	GGAAGTAAAAGTCGTAACAAGG
	ITS2	GCTGCGTTCTTCATCGATGC

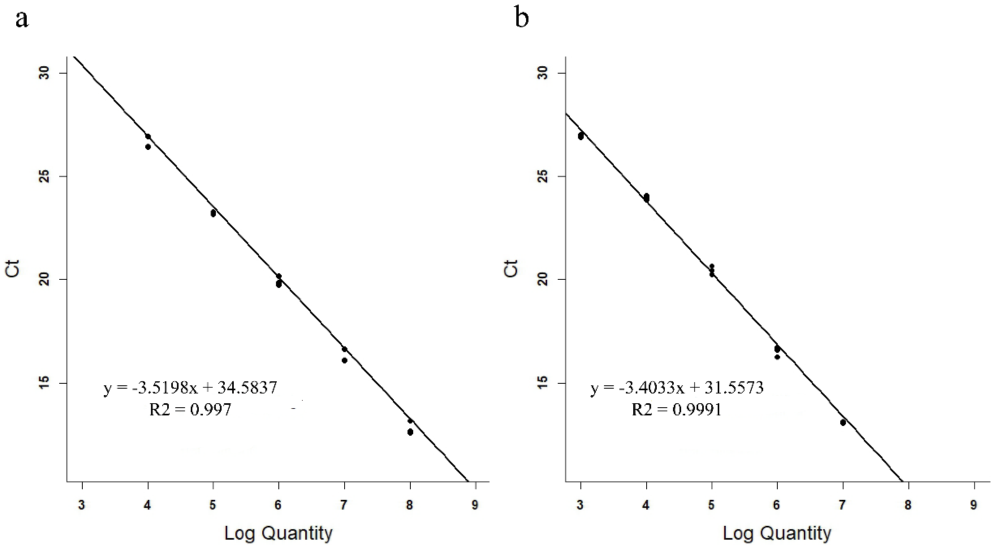

Figure S1. Standard curves of 16S (a) and ITS (b).
